# New directions for complex systems in contemporary neuroscience: a morphodynamic and emergent function approach

**DOI:** 10.3389/fncom.2026.1800523

**Published:** 2026-06-05

**Authors:** Enver M. Oruro, Grace E. Pardo, Alberto A. Rasia-Filho, Norberto Garcia-Cairasco

**Affiliations:** 1 Neurocomputing, Social Simulation and Complex Systems Laboratory, Scientific Research Institute, Andean University of Cusco, Cuzco, Peru; 2 Morphodynamic Neuroscience and Behavior Laboratory, UNESCO UniTwin CS-DC, Cusco, Peru; 3 Neuroscience Research Laboratory, Scientific Research Institute, Andean University of Cusco, Cuzco, Peru; 4 Department of Basic Sciences/Physiology and Graduate Program in Biosciences, Federal University of Health Sciences of Porto Alegre (UFCSPA), Rio Grande do Sul, Brazil; 5 Graduate Program in Neurosciences, Federal University of Rio Grande do Sul (UFRGS), Porto Alegre, Rio Grande do Sul, Brazil; 6 Neurophysiology and Experimental Neuroethology Laboratory, Physiology Department, Ribeirão Preto School of Medicine, Graduate Programs in Physiology and in Neurology, Neurosciences and Neuropsychiatry, University of São Paulo, Ribeirão Preto, São Paulo, Brazil

**Keywords:** brain networks, complex systems, computational neuroethology, emergent functions, epilepsy and comorbidities, morphodynamic neuroscience, neuronal morphology

## Abstract

We propose that current Neuroscience approaches can benefit from further integrating morphodynamics across different scales of brain organization and neural network emergent functions in complex systems. While emergence in neuroscience is commonly addressed at higher organizational levels, here we consider neuronal morphology itself as an emergent level of organization. Progressing from form-based complexity views, early models of neuronal morphogenesis, and functional approaches, we integrate cell morphology to behavior with particular relevance to the following issues: (1) Neuronal Morphological Diversity and Circuitry Function, (2) Mother-Infant Relationships, and (3) Epilepsy and Neuropsychiatric Comorbidities. The structure of neurons and their connectivity within the brain volume are morphodynamic features that emerge from dynamic interactions among morphogenetic elements, the local cell neighborhood, and synaptic connections. In turn, the emergent functions of networks are organized around a series of conceptual, experimental, and computational foundations. Complex systems neuroscience combines such data with additional high- and multiscale information to develop models organized around structure, function, and behavioral displays in both normal and pathological conditions. Here, we present and discuss examples that approximate this framework, drawing on animal models and human data. Such an integrated approach aligns with the ongoing efforts promoted by UNESCO’s “UniTwin Complex Systems Digital Campus” (CS-DC) to collaboratively address open, multiscale problems in neuroscience and complex systems.

## 1 Introduction

Complex systems are characterized by the emergence of multiple levels of structure and organization from interactions among their constituent elements, producing properties that cannot be reduced to the behavior of isolated components. In neuroscience, a central challenge remains understanding how these multilevel interactions give rise to emergent properties across spatial and temporal scales (Gershenson, 2008, 2025). This issue can be examined at higher levels of organization, such as neural circuits, cognitive functions, or population-level behavioral displays, in which the neuron is treated as a basic unit rather than as an emergent structure with its own functional emergent property. Consequently, there is currently no established theoretical or computational framework specifically aimed at understanding how neuronal form itself emerges, is dynamically maintained, and contributes to higher-level functions.

Neurons and glial cells are units of neural circuit complexity, and the properties of complex systems are emergent functions that arise from more than the sum of their parts. That is, while a function relies on the properties of its constituent elements, the organized composite has additional properties when assembled and can support higher levels of activity. Consequently, the emergent properties of a system enable assembled specialized nerve cells to generate and modulate more complex functions and behaviors. In other words, “the isolated parts of an airplane cannot fly, but the whole does fly”; then, “flying” becomes an emergent function (Renner and Rasia-Filho, 2023). In this sense, an emergent function is the property or capability that arises from the organized interaction of multiple elements and cannot be reduced to the isolated properties of the components (Gershenson, 2008, 2025). It is the highly orchestrated organization of these neural units that, together, configure and form the dynamic features of the nervous system across high-order multiscales. This is exemplified by the cytoarchitecture of cortical layers (e.g., with ∼57,000 cells and ∼150 million synapses within 1 mm^3^ of human cerebral cortex; Shapson-Coe et al., 2024), the formation of local microcircuits and major macrocircuits, the spatial organization of neural pathways for sensory, associative, homeostatic, motor, emotional, creative, and cognitive functions. They are integrated to display a multitude of individual and social behaviors in constant interaction with the environment (see further discussion in Rasia-Filho, 2006, Rasia-Filho et al., 2018, 2021). Moreover, nervous tissue is sensitive to various hormonal and interoceptive/visceral influences, some of which also shape cognitive processes such as learning and memory, survival, and adaptation.

Questions concerning the origin and development of morphological complexity in the nervous system, particularly those involving the morphogenesis of nerve cells and the diversity of neuronal shapes, remain insufficiently explored. Neuronal morphology is often considered a static feature after “maturation” and the end of cellular development, rather than a dynamically maintained level of organization with both stable and plastic properties. This limits our ability to understand how neuronal form adapts to experience and contributes to emergent neural functions across individuals and social contexts. It is an ongoing endeavor to reveal how single neurons in the entire nervous system, or in parts of it, morphologically adapt to different experiences, while accounting for intra-individual and inter-individual variability. Addressing this gap requires a conceptual framework that treats neuronal shape itself as a product of interactive processes. To this end, we draw conceptual inspiration from the framework of morphogenesis, proposed by Lesne and Bourgine (2011) to adopt a complex systems perspective. Although their work does not focus on neurons, it provides a general theoretical approach for studying how shapes and patterns emerge from the interaction of morphogenetic elements.

Building on this perspective, we adapt morphogenesis to neuroscience by proposing that neuronal morphology constitutes an emergent level of organization arising from the coordinated interactions among its morphogenetic components. In this view, neuronal morphology is not assumed *a priori* as a final form but rather emerges continuously from the interplay of intracellular processes that shape and reshape the cell. Indeed, early computational approaches to neuronal morphogenesis, such as those developed by Hely et al. (2001), already exemplified this perspective. From this perspective, a growing body of empirical evidence on neuronal development and synaptic plasticity can be interpreted as reflecting underlying morphodynamical processes.

While morphogenesis captures how neuronal form changes in response to morphogenetic determinants, it does not fully account for the continuous modification of neuronal structure across development and beyond, in response to increases or withdrawal of external and internal stimuli, and due to experience. Neural morphology and connectivity emerge through the interplay of genetic determination, gene regulatory networks, and activity-dependent processes, including synaptic overproduction, stabilization, remodeling, and pruning (Rakic et al., 1986; Li et al., 2026; Petanjek et al., 2011). These processes begin during fetal development, where early synaptogenesis contributes to the initial organization of neural circuits through presynaptic specialization and postsynaptic protein synthesis, transport, and assembly (Kostović, 2024). The early appearance of “pioneering” synapses likely plays a morphogenetic role in establishing the fundamental circuitry underlying spontaneous and evoked activity prior to postnatal experience (Kostović, 2024). Structural plasticity is particularly pronounced during development, but synaptic reorganization continues across postnatal stages, including infancy, adolescence, adulthood, and old age, allowing environmental inputs and additional ontogenetic factors to reshape neuronal morphology and circuit function (Petanjek et al., 2011; Bagarinao et al., 2020; Petanjek et al., 2023). For example, synaptogenesis in human cortical areas begins prenatally and extends into early postnatal life, followed by region-specific synaptic elimination during later developmental stages (Huttenlocher and Dabholkar, 1997).

Neuronal morphodynamics is central to the establishment, maintenance, and adaptation of neural connectivity, linking structural plasticity to functional outcomes across multiple temporal scales, as exemplified by early thalamocortical connectivity and sensory-driven structural remodeling in both developing and mature brains (Kostović, 2024; Martin et al., 2022, see also additional references therein). When altered, these processes contribute to neurodevelopmental circuit disorders (Petanjek et al., 2023; Kostović, 2024). Converging evidence from human studies, experimental models, and organoid systems further supports that neuronal structure, connectivity, and activity co-evolve over time, enabling the study of developmental mechanisms and disease-related phenotypes across molecular, cellular, and network levels (Antunes et al., 2025; Tenreiro and Muotri, 2026; Li and Knoblich, 2025; Perets et al., 2026).

Relationship between structure and function can also be addressed on the basis of the work of Luhmann and Molnár, which shows how developmental processes such as neuronal migration, early spontaneous activity, transient circuits, and thalamocortical connectivity participate in the progressive organization of the developing cortex, while function is gradually refined through these early developmental dynamics and interactions (Luhmann et al., 2016; Molnár et al., 2020; Molnár and Kwan, 2024). On a broader level, these relationships can also be considered from an evolutionary perspective. For example, by examining the transitions from three-layered cortices to the six-layered neocortex in the context of environmental and behavioral adaptations that imposed new functional demands on pallial organization, as proposed for the expansion and reorganization of cortical systems in mammals relative to other amniotes (Shepherd, 2011; Aboitiz and Montiel, 2015; Montiel and Molnár, 2013). More generally, this relationship between structure and function can also be extended to frameworks of self-organization and to the notion of dissipative structures, in which spatial and structural patterns arise from non-equilibrium dynamic processes (Prigogine, 1978; Tabony, 1994).

We therefore introduce morphodynamics to emphasize that neuronal morphology evolves differently over time through ongoing interactions with the local cellular microenvironment and spatial constraints, including other neurons, glial cells, intrinsic and extrinsic circuits, and vasculature (Oruro and Pardo, 2025). Morphodynamics refers to organized, regulated changes in cellular structure, combining genetic signals with developmental processes to adapt cellular shape to functional demands. It thus offers a conceptual space for integrating experimental and theoretical micro- and macroscale data across multiple cellular and spatiotemporal scales. In the nervous system, data range from molecular composition and intracellular organization to neuronal morphology, connectivity, and functional integration over time. By placing neuronal morphology—along with its synaptic capabilities to integrate inputs and generate action potentials—at the center of this integration, we propose an additional framework in which the unit of neuronal form enables functional emergence in a complex system, beginning at the cellular level and advancing to networks and behavioral displays.

**FIGURE 1:**
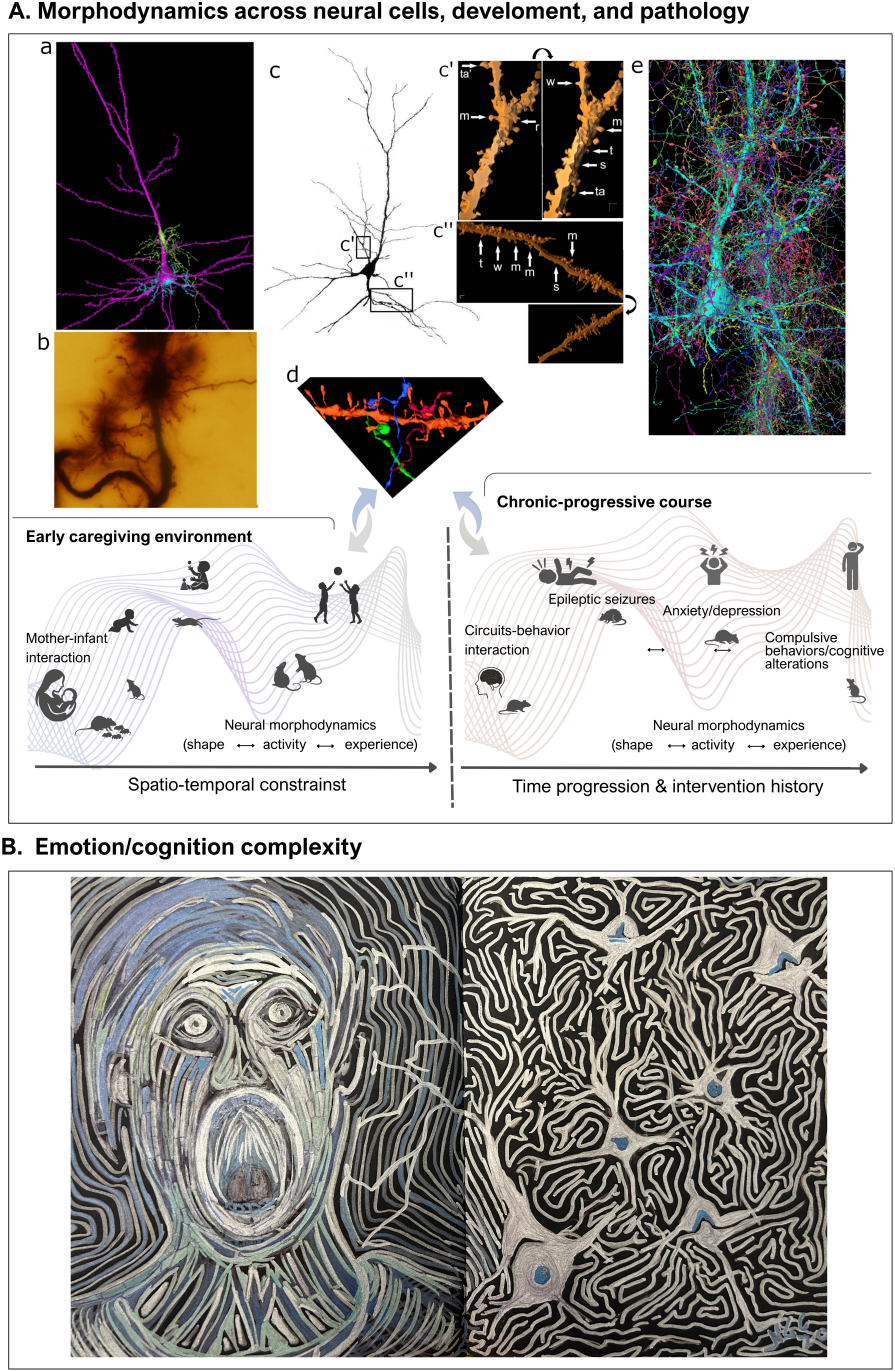
Morphodynamics across neural cells, development, and pathology. **(A)** Human neural cells illustrating the basis of morphodynamics and complex systems, from nerve cells to neural circuits to gain insights into normal and disordered brain function. Upper panel: (a) A cortical layer III pyramidal neuron (magenta) with two close glial cells (microglia in blue and yellow-green). Cells were imaged using high-throughput serial-section transmission electron microscopy (TEM), followed by three-dimensional (3D) reconstruction. (b) Golgi-impregnated protoplasmic astrocytes (under light microscopy) in close vicinity and projecting processes to a local blood vessel, integrating cellular functioning and metabolism with the dynamic control of the brain vasculature. (c) 3D-reconstructed Golgi-impregnated pyramidal neuron (light microscopy) showing the presence, distribution, and shape of dendritic spines (at higher magnification and observation angles in the inserts c’ and c”). Spines were classified as stubby (s), wide (w), thin (t), mushroom-like (m), ramified (r), or transitional/atypical ones (ta, currently named “multimorphic,” with a spinule indicated graphically by an apostrophe). (d) 3D-reconstructed TEM images evidencing multiple connections of different axons (green, blue, and pink) with a short dendritic shaft and spines (orange). (e) Note the huge connectional pattern of one layer V pyramidal neuron (light blue) surrounded by incoming axons (other colors). Consider the geometry of this neuron, the multiple synaptic demands, and temporospatial input codes received by such a spiny dendritic arbor from neural networks. Images a, d, and e (with no changes) are from the left anterior temporal lobe of an adult woman, using the original data published and made available by Shapson-Coe et al. (2024) at https://h01-release.storage.googleapis.com/landing.html (Neuroglancer), licensed under CC BY 4.0. b and c are from the medial and basomedial amygdaloid complex nuclei of adult men [b, obtained in accordance with ethics procedures described in Dall’Oglio et al. (2015); c, reprinted and adapted with permission from Rasia-Filho et al. (2021), licensed under CC BY 4.0]. Lower left panel: conceptual figure illustrating early development as a continuous, history-dependent process across spatiotemporal constraints. Early caregiver environment, mediated primarily through mother-infant interactions, exerts a strong influence during postnatal development with lasting effects. Neural morphodynamics evolve continuously rather than through discrete stages. Changes in neural morphology, functional activity, and behavior co-emerge through reciprocal interactions, giving rise to increasingly complex behavioral and cognitive patterns. Computational modeling and simulation are highlighted as practical approaches for exploring how multilevel emergent phenomena can arise from interactions across time and space. Note emergence across levels of neural organization, and morphodynamics as a complementary analytical approach for studying early development in a complex systems perspective. Lower right panel: Epilepsy is a chronic, progressive condition emerging from distributed, multiscale interactions among neural circuits, behavior, and experience over time. Neural morphodynamics continuously shape and is shaped by circuit-behavior interactions along the disease course. Note that, rather than arising from isolated focal dysfunctions, epileptic seizures and associated neuropsychiatric comorbidities are depicted as emergent pathological dynamics conditions occurring along partially overlapping trajectories. The temporal axis integrates disease progression with the cumulative history of interventions, emphasizing history-dependence and the non-linearity of pathological emergence. Again, computational modeling and simulations are useful to unravel how organizational emergence arises from distributed interactions across organizational levels. **(B)** Complexity of brain networks associated with emotional and cognitive events to artistically illustrate the continuum of morphogenetics to complex system functioning. Artwork reproduced with permission from NGC-2023, sharpie color pens in black paper, inspired by “The Scream” (1910) by Edvard Munch.

**FIGURE 2:**
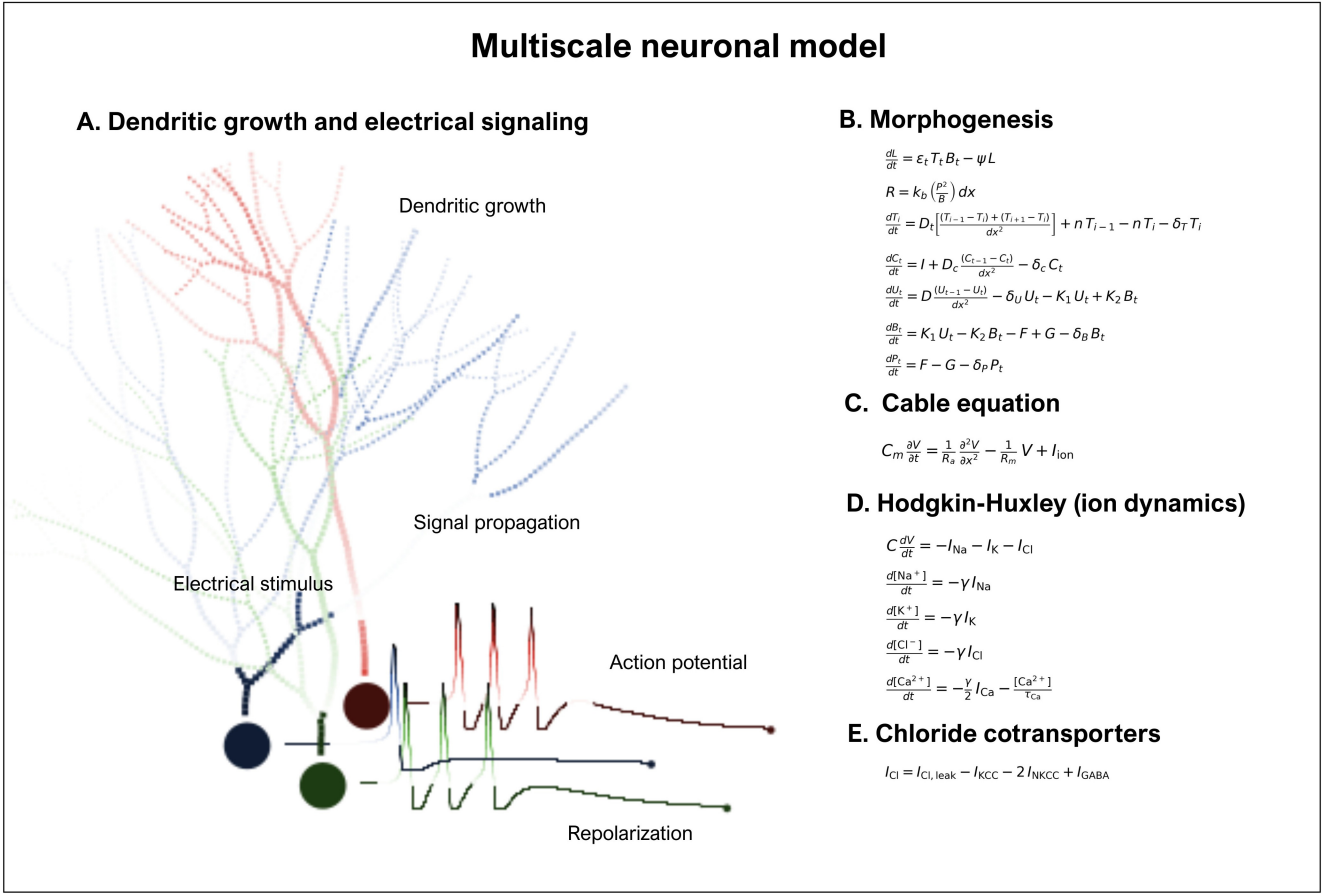
Multiscale neuronal model integrating dendritic morphogenesis, cable propagation, and Hodgkin-Huxley dynamics with dynamic ion concentrations. **(A)** Morphogen diffusion is represented by the progressive dendritic growth of three neurons (blue, green, and red). Once the dendritic arbors are established, the neurons receive an electrical stimulus at a random location, depicted as a fading white shadow passing through the dendrites. The passage of this white shadow represents dendritic depolarization, which propagates along selected activated pathways toward the soma. As the depolarization wave passes, the branches become dark, indicating repolarization. This process can eventually trigger somatic action potentials, after which the membrane repolarizes and hyperpolarizes. Soma color also varies as a function of somatic voltage: during an action potential, the soma changes from dark to white and then to a more intense red, blue, or green, depending on the neuron, indicating neuronal firing. The corresponding action potential traces are displayed in the same color as each neuron. Afterward, during hyperpolarization, the soma returns to darker tones. In this color code, white indicates membrane potentials above resting levels. This sequence is also shown in the accompanying simulation (Supplementary Video). **(B)** Dendritic growth is governed by morphogenetic equations based on tubulin, MAP-2, and calcium dynamics, including elongation, branching probability, and intracellular transport across compartments. **(C)** Electrical propagation is described by the cable equation defined over the generated dendritic morphology. **(D)** Membrane dynamics follow a Hodgkin-Huxley formalism with time-dependent ion concentrations for Na^+^, K^+^, Cl^–^, and Ca^2+^. **(E)** Chloride homeostasis is regulated by KCC2 and NKCC1 cotransporters, which contribute to the net chloride current and modulate GABAergic signaling. Complete equations for B are described in Oruro and Pardo (2025), and equations for D and E are described in Becerra-Flores et al. (2026). Simulation of the model was made using NetLogo 7.0.4 (Wilensky, 1999; http://ccl.northwestern.edu/netlogo).

Here, morphodynamics is a unifying conceptual framework that links neuronal morphology, circuit functions, and behavior across multiple spatial and temporal scales. We propose that neuronal morphology can be understood as an emergent and continuously evolving level of organization, shaped by interactions among cellular components/morphogens, neighboring cells, spatial constraints, and environmental signals. This perspective holds across both normal and pathological conditions and provides a basis for integrating diverse data streams into computational and theoretical models of nervous system organization and function (Figures 1A, 2).

## 2 Morphology and emergent property organization

We examine how neuronal morphology can be understood as an emergent level of organization within a morphodynamic perspective, linking cellular structure with circuit complexity and functional outcomes. In this section, we focus on how morphogenesis and morphodynamics contribute to the organization of neuronal form and its role in shaping neural circuitry and emergent functions.

Neuronal morphogenesis and morphodynamics are key processes to consider in the functional specialization of the nervous system and in complex systems research. Let us consider that the biochemical and biophysical features of neurons and glial cells can be studied at deeper, more intricate levels to determine whether emergent properties of intracellular molecular interactions arise. On a higher scale, let us also consider what happens to neuronal morphology and its role in circuitry complexity.

Neurons undergo continuous transformations across development, as dendrites elongate, branch, stabilize, or retract in response to genetic factors, local neurotrophic factors, connectivity and function, and environmental signals. Neurons differ in how their dendrites branch within the neuropil volume and how they receive inputs from different afferent sources along their arbor (Cajal, 1909–1911; Ramon-Moliner, 1962; Yuste, 2010; Renner and Rasia-Filho, 2023). In turn, the shape of a neuron can actively influence other neighboring cells and be influenced by the further development of micro- and macrocircuits, generating reciprocal morphodynamic interactions. Each time a neuron increases and spreads its arbor, it not only imposes additional biophysical features that affect its function (Spruston et al., 2013) but also occupies a particular space in a restricted species-specific brain volume. This is a dynamic, adjustable process that demonstrates experience-dependent structural plasticity. The intracellular calcium and the actin and microtubule cytoskeletal architecture are critical for numerous physiological conditions involving synaptic transmission, long-term potentiation, and long-term depression, whereas their dysfunction relates with various neurodegenerative and psychiatric diseases (Alzheimer’s disease, Parkinson’s disease, amyotrophic lateral sclerosis, tauopathies, and prion disorders; Rakovskaya et al., 2026). Further linking neuronal shape and function, sensory deprivation delays the maturation of dendritic and receptive fields in retinal ganglion cells in the visual system (Chen H. et al., 2021) and alters the differentiation of primary and secondary dendrites of mitral cells in the olfactory system (Matsutani and Yamamoto, 2000).

In many neurons, dendritic spines, tiny cellular specializations that span varied shapes, sizes, and clustering patterns, increase neuronal connectivity, synaptic plasticity, and functional features (Arellano et al., 2007; Bourne and Harris, 2009; Yuste, 2010; Rasia-Filho et al., 2023a,2023b; Renner and Rasia-Filho, 2023; Renner et al., 2026a,b). Spines modulate the “weight” of most postsynaptic excitatory potentials (but also inhibitory or other modulatory actions), providing neural networks with high integrative and computational capabilities, circuit stability, as well as plasticity for information processing depending on the synaptic demands (Yuste, 2010; Kubota et al., 2016; Rasia-Filho et al., 2023a; Harris et al., 2024). Some newly formed spines become stable, whereas the number of other plastic dendritic spines can change due to different causes and time scales (reviewed in Petanjek et al., 2023; Rasia-Filho et al., 2023a,b). In this regard, synaptic pruning happens mostly on spines in the human prefrontal cortex (Petanjek et al., 2011, 2023; see also Kostović, 2024), and reduction in the number of dendritic spines and synapses reorganize and mature circuits ontogenetically (Petanjek et al., 2023). Plastic spines allow circuits to adapt their function to new experiences; increased spine density provides more synapses and enhanced information processing when necessary. Demonstrating a clear cause-and-effect relationship, Hayashi-Takagi et al. (2015) used optogenetic manipulation to promote the shrinkage of potentiated spines and, at the same time, erase specific synaptic memory traces in the mouse motor cortex. However, some neuroplastic processes can be deleterious when the triggers are, for example, traumatic injuries, ischemic-hypoxic processes, or seizures, where cell proliferation (neurogenesis) is ectopic and axonal sprouting can reverberate in uncontrolled hyperexcitability. A neuroinflammatory environment worsens this scenario in epilepsies or neurodegenerative diseases (Servilha-Menezes and Garcia-Cairasco, 2022; Ramos et al., 2025).

Human spines exhibit site-specific differences compared with their mouse counterparts (e.g., they are comparatively larger and longer in CA1 pyramidal neurons), indicating evolved features in synaptic processing and system organization (Benavides-Piccione et al., 2020; Rasia-Filho et al., 2023a; Benavides-Piccione et al., 2025; Figure 1A, upper panel). Hence, new questions are raised to understand how form and function develop together (Renner et al., 2026b) and how connections are processed and stabilized across temporospatial scales to generate emergent properties. Computational models have been developed to represent morphogenesis and neural morphology, including the variety of shapes within or among different types of neurons, growth during development, and the influence of social stimulation. They can also address, for example, neuroplasticity changes in experimental epilepsy models (with neurodegeneration/neuroinflammation, cell proliferation, and network rearrangements) and their clinical equivalents, such as neurodegeneration, reactive neurogenesis, and hippocampal mossy fiber sprouting. Such integrative efforts can guide the design of morphology-focused experiments in complex systems neuroscience. In this regard, the UNESCO UniTwin Complex Systems Digital Campus (CS-DC) addresses and integrates current efforts grounded in experimental data to promote new research and education strategies, with significant translation value, in complex systems science.^1^

In the following sections, we discuss the challenges that have arisen along this integrative research pathway, opening new avenues for investigating neuronal morphology, early development and behavior, epilepsies, and their associated neuropsychiatric comorbidities, as well as providing key information for integrating these domains into computational models useful for complex systems science.

## 3 The development of morphological features for the function of complex systems

Neurons and glial cells, their cytoarchitectural organization and connectivity patterns, and the formation of both local and long-range neural networks are morphologically and functionally heterogeneous across phylogeny and ontogeny (Figure 1A, upper panel; Cajal, 1909–1911; DeFelipe, 2011; Renner and Rasia-Filho, 2023). Evolved brain functions include (but are not limited to) the emergence and development of main cellular types, such as pyramidal neurons and a variety of local interneurons, in hub areas in the subcortical-allocortical-neocortical *continuum* related to emotional and social information processing, higher-order associative processing, and cognition (Cembrowski and Spruston, 2019; Kolb and Whishaw, 2021; Rasia-Filho et al., 2021). Humans have a particular cerebral cortex neuropil structure, connectional pattern, and cellular functioning (DeFelipe, 2011; Eyal et al., 2016; Schmidt and Polleux, 2022; Kanari et al., 2025). For example, relevant functional specializations in humans arose from further development of biophysical properties in dendrites, connections and their synaptic strengthening along with the expansion of layers II and III for processing cortico-cortical circuits (Eyal et al., 2016; Planert et al., 2025). Differently shaped dendritic spines are also associated with high intracellular molecular diversity (Ammassari-Teule et al., 2021; Helm et al., 2021), thereby increasing the repertoire for signal elaboration and transduction associated with more complex neuronal functioning.

In addition, human synapses have (1) stronger connections, related to larger presynaptic active zones and postsynaptic densities, (2) active zones that have a shape and size allowing for a more readily releasable and recyclable pool of synaptic vesicles, and (3) higher neurotransmitter release, binding, receptor family subunits, and signaling compared to other studied animals (Yakoubi et al., 2019a,2019b; Rollenhagen et al., 2020; Kalmbach et al., 2021; Hunt et al., 2023). The unique intrinsic electrophysiological membrane properties of human cortical neurons amplify synaptically induced depolarizations and communication from dendrites to the soma and axon (Eyal et al., 2016; Hunt et al., 2023). This results in cortical region- and layer-specific features involving morphological and functional crosstalk among neuronal dendritic arbor architecture, synaptic structure, and neurotransmission strength (Yakoubi et al., 2019a,2019b; Rasia-Filho et al., 2021). Excitatory synaptic boutons in the human cortical layer IV may also act as “amplifiers” of signals from the sensory periphery to integrate, synchronize, and modulate intra- and extracortical synaptic activity (Yakoubi et al., 2019b). These data exemplify how system organization complexity increases from cellular structural arrangements to the functional specializations of brain areas and neural circuits, i.e., from cell composition, morphodynamics, and network-integrative roles to advanced neural computational capabilities and behaviors (Figure 1, upper panel).

Going one step further, morphological features of dendrites and spines enable neurons to modulate multiple synaptic inputs in different domains (Renner and Rasia-Filho, 2023). By integrating information processing across different spatiotemporal scales, the human brain uses local microcircuits and major networks to modulate the dynamic activity of cells within each interconnected area and to support multiple, hierarchical, and parallel functions. Changes occur throughout the lifespan in the brain’s short-range, intrinsic connectivity and in the long-range, overall connection strength across the entire brain (Bagarinao et al., 2020). For the latter, the default mode, the salience detection, and the frontotemporal and frontoparietal circuits involve coordinated activities for attention, self-monitoring processes, cognitive leisure and exertion, and emotional processing (Kolb and Whishaw, 2021; Schimmelpfennig et al., 2023). The great limbic lobe evolved from some subcortical amygdaloid nuclei into allocortical and neocortical regions, including multimodal associative areas, to enhance emotional perception and the display of more elaborate social behaviors (Heimer et al., 2007; Panksepp, 2016; Diano et al., 2017; Rasia-Filho et al., 2021). One such evolved capability is the interpretation of facial expressions with social valence in primates (Rutishauser et al., 2015). In this regard, the occurrence and development of specialized pyramidal neurons with a thicker and variably long apical dendrite, along with two or more branched basal dendrites, provided greater computational and functional capabilities in such a neural network (Rasia-Filho et al., 2021). Long and branched dendrites offer a more extensive receptive field while establishing dendritic domains relevant for the distribution of different input connections (Larriva-Sahd, 2014; Dall’Oglio et al., 2015; Renner and Rasia-Filho, 2023). Passive and active dendritic properties influence the amplitude and duration of linear and non-linear postsynaptic potentials, depending on the location and strength of these synaptic inputs (Spruston et al., 2013). A variety of interneurons provide local inhibition to pyramidal neurons, modulating cortical action-potential output patterns in neural networks. Morphology is, again, a key point for understanding function and emergent complex behavior; together, they identify the minimal structural, active, and interactional components required to model the emergence and maintenance of neuronal form under ontogenetic and evolutionary constraints within a morphodynamic framework.

### 3.1 Morphological heterogeneity is part of complex systems functioning

Morphological heterogeneity is the rule within and among neuronal and glial cell types (Cajal, 1909–1911; Oberheim et al., 2009; Cembrowski and Spruston, 2019; Rasia-Filho et al., 2021; Guerra et al., 2023). Shape and function are closely related, indicating multiple information-processing and activity ranges within assembled, morphologically distinct neural cells. Indeed, (1) although belonging to the same class, spiny pyramidal neurons in layers II/III of the human temporal cortex are electrophysiologically heterogeneous and form four subtypes of cells, corroborated by differences in morphology (Planert et al., 2025). And, (2) human neurons vary from the aspiny or “relatively aspiny” cells in the spinal cord—likely optimized to “direct” synapses on dendritic shafts for fast, protective stereotyped reflex responses—to subcortical, hippocampal, and neocortical neurons, which are usually covered with a low-to-moderate, high, or even very high density of clustered pleomorphic spines—involved with multiple routes for synaptic modulation and more complex neural elaborations— (Yuste, 2013; Vásquez et al., 2018; Rasia-Filho et al., 2021, 2023a,2023b; Renner and Rasia-Filho, 2023). Multiple synaptic processing in spiny dendritic trees results in greater signal integration, biophysical and biochemical compartmentalization, synchronized modulation of current flows, and, eventually, a broader output firing to interconnected circuits (Spruston et al., 2013). Morphological features of spiny dendrites underlie neuron-type-specific connectivity, brain wiring, and input-specific, synaptically induced, complex functional properties within neural networks (Yuste, 2010; Chen et al., 2011; Spruston et al., 2013; Tønnesen and Nägerl, 2016; Kasai et al., 2021; Renner and Rasia-Filho, 2023).

Neuronal shape is related to its structural proteins and functions. The shape, synaptic role, and plasticity of dendritic spines involve various local proteins (Helm et al., 2021; Rasia-Filho et al., 2023a). For example, the mammalian target of rapamycin (mTOR) signaling pathway is associated with spine development and pruning (Chaudry and Vasudevan, 2022). Transcription factors associated with intrahippocampal neurotransmission between mossy fibers with thorny excrescences, a local complex multilobed postsynaptic structure (Wilke et al., 2012), would serve as molecular markers of synaptic plasticity in some multimorphic spines (Renner et al., 2026a). On the other hand, defective proteostasis contributes to synapse dysfunction, memory impairment, and the progression of neurodegenerative disorders, including Alzheimer’s disease (Cozachenco et al., 2023). Abnormalities in the polymerization and remodeling of the actin cytoskeleton and its associated proteins lead to functional consequences resulting in symptoms and clinical correlates of ASD (Falougy et al., 2019).

Neurons work together with glial cells, which are also morphologically larger and show greater microanatomic complexity in the human cerebral cortex (Oberheim et al., 2009). Glial cells functions add to the complexity of the nervous system in a remarkably complex and broad manner (e.g., Grotzinger et al., 2026). Glial cells in the central and peripheral nervous system (1) regulate neuroblast proliferation, migration, neurogenesis, spinogenesis (maturation or shrinkage) and synaptogenesis; (2) promote axon outgrowth, connectional development, myelination and conduction velocity within networks, but also regulate axon pruning, synapse elimination, and programed cell death; (3) modulate synaptic transmission and the availability, turnover, and spillover of neurotransmitters or secrete gliotransmitters; (4) compose the blood-brain barrier, release vasoactive substances, and, when coupled to local blood vessels, modulate the microcirculation (Figure 1A, upper panel); (5) promote ion buffering, water and osmotic homeostasis, regulate the extracellular pH, and allow movements of inorganic and organic molecules; (6) synthesize glycogen and provide lactate to support the neuronal metabolic/energetic demand; (7) adjust neuroendocrine secretion and various behavioral displays, circadian rhythms, sleep, respiration, and pain sensation; (8) are related to local immune and local or systemic inflammatory responses, have phagocytic properties, remove neuronal debris, respond to insults to nervous tissue, repair damage, and produce regenerative responses (Rasia-Filho et al., 2023b and references therein); and (9) compose the glymphatic, meningeal lymphatic, and periarterial spaces network for interstitial waste solutes clearance and immune surveillance (Kipnis et al., 2025). Each one of these actions has implications for the functioning of a complex system. The neuroglial structural and functional organization provides neural networks with greater connectional and integrative possibilities, as well as both stability and higher plasticity to adapt to dynamic synaptic demands (Rasia-Filho et al., 2023b).

Therefore, morphodynamics focuses on how neurons and glial cells are structurally developed to receive and respond to a multitude of connectional inputs, and how they generate different functional outputs, leading to a broad repertoire of actions locally and in long-range neural networks (Figure 1A, upper panel). In fact, this is a crucial step in the complex task of determining the functional features and implications of each cell and brain area, as pieces of a great mosaic (or puzzle). One realizes that neuronal and glial subpopulations, having evolved in distinctly organized circuits, function as a temporally and synaptically coded, responsive orchestra capable of performing various meaningful symphonies. Each node/hub (and there are multiple nodes/hubs with intermingled subsets of cells with distinct temporal dynamics and connectional strengths) receives weighted inputs that are integrated to produce an output code, with the entire system displaying a repertoire of flexible possibilities within the limits of each circuit. At the cellular level, the number, spatial distribution, and strength of each synaptic contact along the extension and branching order of the receptive field are modulated by the interaction of each axon upon dendritic domains and, if existing, also upon pleomorphic spines. Together, the orchestra functions with different input sources and activity-dependent intensity, combined with variable amplitudes of functional output codes from the embedded components. Graded ups and downs in cellular excitability over time indicate that specific cells and areas will be activated, while others will be less activated or even temporarily deactivated. These dynamic switches generate distinct codes that represent the interpretation of data under processing. It is expected that different types of cellular and network computations manifest in different time-series features of coordinated activity gradients (“sounds”) within and between brain areas (Shafiei et al., 2020). Ultimately, these functional codes are established by integrating neural population activity patterns—analogous to different instruments played by musicians in an orchestra. The sequence and intensity of “sounds” performed by each “group of instruments” are determined by the functional connectivity of their elements under refined spatiotemporal fine-tuning, with the “symphony” representing the emergent properties of the orchestra as a system (Renner and Rasia-Filho, 2023). In this same vein are the fundamental scientific and philosophical reflections made by Edelman (1987) in his book *Neural Darwinism-The Theory of Neuronal Group Selection* and by Noble (2008), one of the founders of Systems Biology, in *The Music of Life*. Additional theoretical, experimental, and computational approaches to the morphodynamics properties of the elements and the whole system may include *in vitro, in vivo*, and *in silico* models, studies in normotypic or in neurological and neuropsychiatric conditions—including ASD, epilepsies/comorbidities, mood disorders, or neurodegenerative diseases, some described below (Heimer et al., 2007; Hrvoj-Mihic et al., 2017; Kolb and Whishaw, 2021; Renner and Rasia-Filho, 2023).

To illustrate how morphodynamic frameworks extend beyond cellular and circuit levels, we present early development and the mother-infant relationship as a representative domain in which emergent properties arise from multiscale interactions linking neural structure, function, and behavior. This example allows us to examine how morphodynamics operates across levels–from cellular processes to behavioral and social interactions–within a developmental context.

## 4 Early mother-infant relationship, neural development, and emergent behavioral display

From a morphodynamic perspective, early development can be understood as a multiscale process in which neural structure, function, and experience co-evolve over time. To illustrate how these concepts can be applied beyond neuronal morphology *per se*, we turn in this section to early development and the mother-infant relationship as a representative domain. This field offers a rich experimental and theoretical landscape in which emergent properties arise from multiscale interactions among neural circuits, bodily states, behavior, and social experience across early life.

Attachment is the first social bond between an infant and the primary caregiver (Bowlby, 1982). In the 1950s, Bowlby’s theory offered a pioneering perspective on attachment, integrating biological and psychological approaches, grounded in both clinical work and observations of humans and other animals (Bowlby, 1982). This work established a new paradigm for the study of infant social development and responses to maternal separation (Ainsworth et al., 1978). Bowlby conceptualized attachment as an evolutionary behavioral system that promotes proximity to caregivers for survival (Bowlby, 1982). Subsequent work in developmental psychobiology and behavioral neuroscience provided empirical grounds for attachment as a key concept in early social relationships and neurodevelopment. Research with animal models showed that attachment could be disentangled into relatively independent regulatory systems, such as thermal, nutritional, communicative, and affective, each with its own organizing principle (Hofer, 2004, 2006). Further studies revealed how variations in maternal care affect stress reactivity and behavior, linking early caregiving to long-term neural development of the offspring (Meaney, 2001), and showing how adverse early experiences translate into long-lasting consequences and risk factors for psychiatric disorders in adulthood (Debiec and Sullivan, 2014; Kundakovic and Champagne, 2015; Sullivan and Opendak, 2021). These findings position attachment as a multicomponent regulatory system whose development depends on dynamic interactions across structural and behavioral levels.

Building on this framework, specific neurobiological circuits underlying early attachment formation (Moriceau and Sullivan, 2005), even under adversity (Moriceau and Sullivan, 2006), were identified. Data revealed that mechanisms that ensure attachment can also lead to an infant becoming attached to a threatening caregiver, which occurs during sensitive periods critical for maternal impact on learning and neural plasticity (Moriceau and Sullivan, 2006; Debiec and Sullivan, 2017). Cross-species research has explored the impact of adverse early experiences related to attachment on neurobehavioral developmental outcomes (Curley and Champagne, 2016; Nelson and Gabard-Durnam, 2020; Abramson et al., 2024). However, important gaps remain in understanding how these caregiving elements are dynamically integrated over time within developing neural circuits, the timing of long-term impacts, and the translation of animal findings to the more complex human attachment construct, particularly at the level of developmental processes (Tottenham, 2020; Colombel et al., 2023; Opendak et al., 2025).

To clarify these gaps, it is necessary to specify what development is and how developmental processes relate to neural circuits and the complex emergent functions they support. First, from developmental psychobiology, Michel and Moore (1995) define development as a historical, irreversible process unfolding in open systems through reciprocal organism-environment interactions, rather than as the execution of a predetermined program directed toward adult function (Michel and Moore, 1995). Developmental change is inherently non-linear, multilevel, and history-dependent, giving rise to emergent outcomes through ongoing interactions among genetic, neural, behavioral, and environmental factors (Michel and Moore, 1995). That is, attachment emerges as a multilevel phenomenon encompassing brain, cognition, behavior, and social context (Bowlby, 1982; Hofer, 2004), scaffolded by dynamic developmental resources and shaped by the dynamic mother-infant relationship (Moore, 2007; Lucion and Bortolini, 2014). From this perspective, attachment can be understood as a complex system in which emergent properties at each level cannot be explained in isolation, but only by tracing development over time through organism-environment interactions (Michel and Moore, 1995; Karmiloff-Smith, 2009). In this context, complexity is a property of the developmental process under study that requires explicit modeling of interactions across time and space. This approach motivates integrative and computational approaches to link mother-infant interactions with biological processes across gene expression, neurons, circuits, and behavioral scales.

Nevertheless, a conceptual limitation remained regarding the specific developmental processes through which early experiences shape later outcomes, as well as how altered trajectories occur during development (Gunnar, 2020; Karmiloff-Smith, 2009). In most cases, pathological outcomes observed in adolescence or adulthood are treated as endpoints, without addressing whether and how earlier deviations may have emerged at different levels of organization during infancy (Karmiloff-Smith, 2009). From a dynamic perspective, this reflects the absence of frameworks capable of capturing development as a continuous, history-dependent process unfolding within spatiotemporal constraints (Michel and Moore, 1995; Cainelli et al., 2025; Karmiloff-Smith, 2009). Here, we highlight that our morphodynamic perspective should be considered a complementary analytical approach to investigate the dynamic interactions among neural form, function, and experience.

Therefore, if early development can be understood as a dynamic interplay between the organism and its environment, with the caregiver as a primary mediator, developmental changes cannot be fully captured by isolated molecular, synaptic, or behavioral measurements. Instead, such changes unfold moment to moment through interactions that simultaneously shape neural cells, their function, and, ultimately, behavior. During early brain development, molecular, electrophysiological, and large-scale morphological transformations co-occur with experience, as cortical architecture and dendritic arbors are being organized (Poo and Isaacson, 2007; Pardo et al., 2018; Moreno-Velasquez et al., 2020; Oruro et al., 2020a). That is, infants engage with their caregivers while both neural architecture and behavioral capacities are still being formed. This process is bidirectional: neural development is guided by genetic programs and molded by stimuli, while caregiver behaviors adapt in response to the infant’s interactions (Moore, 2007; Lucion and Bortolini, 2014). Altered caregiver environments affect development by acting upon a dynamically evolving morphological landscape, rather than acting on a fixed substrate (Figure 1A, lower left panel). This is a valuable example of plasticity for the emergence of relevant behavior in a complex system.

While many studies quantify changes in maternal care under early adversity, often reducing them to indices such as entropy that capture unpredictability (Davis et al., 2017; Baram et al., 2012), such measures fail to capture the temporal organization of interactions. From a developmental dynamic perspective, the processes often lost are those through which maternal experience continuously influences multiple levels of the infant’s nervous system, allowing cellular structure, activity, and experience to co-evolve over time. This perspective can help specify the type, frequency, and intensity of experiences to which developing neural circuits are sensitive during early development, thereby delineating emergent trajectories associated with later phenotypes. Addressing these questions requires computational modeling to test hypotheses about emergent properties across levels and scales, enabling the reconstruction of developmental trajectories from discrete empirical observations. In this context, computational approaches, including agent-based models, provide a powerful way to represent interactions and emergent properties (Figure 2). Indeed, classical developmental models have shown that early collective behaviors, such as huddling in neonatal rats, emerge through self-organizing interactions over time and space without centralized control (Schank and Alberts, 1997, 2000). By grounding such models in known mechanisms of neural growth and plasticity and in a specific type of experience, it becomes possible to explore how cumulative interactions among morphology, physiology, and environment give rise to divergent developmental outcomes.

Accordingly, one line of work in this direction is our computational modeling and simulation of the infant olfactory circuit, integrating cellular and synaptic data to explain why maternal odor learning occurs during a sensitive period and why it ends afterward (Oruro et al., 2020a,2020b; Figure 2). This approach illustrates how developmental trajectories can be reconstructed from process-level dynamics, rather than inferred from endpoints, to account for the emergent capacity of the circuit to learn maternal odor during a specific developmental window. By modeling the electrophysiological properties of pyramidal neurons in the anterior piriform cortex during the sensitive and post-sensitive periods of attachment learning in infant rats, we showed that both passive and active membrane properties support rapid acquisition of a conditioned odor during the sensitive period, whereas the progressive maturation of these properties reduces this learning capacity over time (Oruro et al., 2020a). At the behavioral level, the model also accounts for documented phenomena in infant attachment learning. During conditioning, the unconditioned stimulus does not elicit an immediate conditioned response, whereas odor-guided approach behavior emerges during subsequent testing. The simulations indicate that this behavior may not arise *de novo* but rather reflects the recruitment of pre-existing associative mechanisms. Prior to experimental conditioning, infants have already learned the maternal odor through a similar associative process, engaging a neuronal population that mediates orientation and approach toward the caregiver. During the sensitive period, conditioning a novel odor with stimuli mimicking maternal care recruits additional odor-responsive neurons that support approach behavior. As development proceeds, the progressive maturation of these neuronal populations alters their responsiveness, reducing their recruitment by new conditioned stimuli and, thereby, contributing to the closure of the sensitive period (Oruro et al., 2020a). Also, the highly developed ability to learn conditioned odor at the circuit level was explained by integrating GABAergic synaptic transmission data into the model, in which GABAergic input acts to amplify the odor-sensory driving activity in pyramidal cells during the sensitive period of attachment learning (Oruro et al., 2020b).

To provide further perspectives to study morphodynamics, a morphogen-based model was developed to simulate and test the relationships between structural proteins, intracellular signals, and their impact on neuronal morphology quantitatively, as modified by environmental/social behavioral experience (Oruro and Pardo, 2025; Figure 2). Based on the morphogenesis of pyramidal neurons in the anterior piriform cortex, we also developed an agent-based computational model grounded in experimental morphological data obtained during the sensitive and post-sensitive periods of attachment learning in rodents (Moreno-Velasquez et al., 2020). The model simulates neuronal development from P0 to P7 and subsequently to P14 through an agent-based model based on reaction-diffusion dynamics of intracellular calcium levels, tubulin, and microtubule-associated protein 2 (MAP-2), which give rise to dendritic elongation and branching patterns (Oruro and Pardo, 2025). This approach focused specifically on morphogenetic processes, capturing how intrinsic cellular interactions can account for the progressive structural elaboration of pyramidal neuron shape during early development. From this study, we realized that this morphogenetic model did not reproduce the complete neuronal shape that emerges over time within a limited developmental window, suggesting that additional levels of interaction need to be integrated to study the dynamic interplay between neuronal form, function, and experience. In other words, we aim to move from morphogenesis to morphodynamics, incorporating additional relevant elements that are yet to be discovered.

Another approach examined altered caregiving behavior using the “limited bedding and nesting” (LBN) paradigm during the first postnatal week, a sensitive period for attachment learning (Pardo et al., 2024). Under LBN conditions, maternal behavior displays altered frequency and transition patterns (Pardo et al., 2024, 2025). Although these alterations do not induce impairment in attachment-related behaviors on postnatal (P) 15 rats (Pardo et al., 2025), the infants show reduced protein levels of KCC2 (2 K + /Cl-cotransporter; a chloride extruder) and NKCC1 (Na-K-2Cl cotransporter; a chloride importer) in the anterior olfactory cortex, a structure necessary for attachment learning in infant rats (Colombel et al., 2023). To examine the functional consequences of these molecular changes, a computational model of pyramidal neuron activity was developed using a Hodgkin-Huxley model with realistic ion concentration dynamics (Lemaire et al., 2021), incorporating KCC2 and NKCC1 expression data from LBN-exposed infants. Simulations revealed that these alterations modify neuronal responses to GABAergic inputs, leads to increased activity compared to controls and suggest a profile of activity characteristic of the sensitive period (Becerra-Flores et al., 2026).

The lasting effects of maternal behavior can also be examined under conditions of social and environmental adversity. Several experimental paradigms evoke the effects of reduced maternal stimulation or altered quality of care, such as the maternal separation protocol and conditions associated with fragmented and unpredictable caregiving, including the “LBN” paradigm (Baram et al., 2012; Glynn and Baram, 2019). Other scarcity-adversity paradigms have been associated with abusive caregiving profiles (Roth and Sullivan, 2005; Perry et al., 2019). By considering the temporal organization of mother-infant interactions as a relevant condition of experience, we applied network analysis to caregiving transitions under LBN conditions, capturing maternal care as a dynamic process that can be linked to pup development (Pardo et al., 2024, 2025). Importantly, pup signals also dynamically modulate maternal responses under adversity (Lapp et al., 2024). These behavioral analyses provide a description of the temporal organization of mother-infant interactions, offering further frameworks for identifying experience-derived signals that may be relevant to shaping neural development.

Taken together, these examples highlight how early development can be understood as a continuous, history-dependent process in which neural, behavioral, and environmental interactions give rise to diverse developmental trajectories. Across different levels of analysis, they show how moment-to-moment patterns of caregiving, circuit-level dynamics, and cellular properties can be examined to reconstruct developmental trajectories, rather than being inferred from isolated endpoints. Applying a morphodynamic perspective to experimental data on a given developmental phenomenon across multiple levels of organization offers a way to investigate the emergence of properties in the developing organism and its nervous system, from cellular morphology to circuit-level dynamics and from individual behavior to social interactions. Exploring how form, function, and experience dynamically interact over time, and how early experiences shape enduring brain, behavioral, and cognitive trajectories, currently represents a central challenge in developmental psychobiology and the study of the mother-infant relationship.

We next extend the morphodynamic framework to pathological conditions, using epilepsy and its neuropsychiatric comorbidities as a representative case. This section illustrates how alterations in neuronal morphology, circuit organization, and multiscale interactions contribute to emergent dysfunctions in complex neural systems. By examining epilepsy within this perspective, we aim to highlight how structural and functional changes are intertwined across levels, from cellular processes to behavioral manifestations.

## 5 Neural circuits, epilepsy, and neuropsychiatric comorbidities

From a morphodynamic perspective, epilepsy can be understood as a disruption of multiscale interactions that normally support stable and adaptive neural function. In the search for brain mechanisms associated with epilepsies—which were historically associated with sacred concepts of potential “demons’ possession” or divine blessings (Fadiman, 1997; Eadie and Bladin, 2001)—we have reached an understanding of the role of neural cell morphology and relevant molecular mechanisms causing abnormal activity in hypercomplex sets of parallel and redundant circuits (Figure 1A, lower right panel). These networks display multiple protagonist elements organized across various scales involved in both normal brain function and behavior, as well as in the abnormal findings of epilepsies, with emphasis on pharmacoresistant ones. Conceptual and technical developments in epilepsy research revealed an additional level of complexity by considering the usual and associated neuropsychiatric comorbidities at several ages, such as the ASD (in children); anxiety, depression, and compulsive behaviors (in young adults), and Alzheimer’s disease (in elderly people; Servilha-Menezes and Garcia-Cairasco, 2022; Alves et al., 2026 **).** A detailed description of “symptom network analysis” of comorbidities in epilepsies was presented by Gauld et al. (2021). In this regard, ongoing protocols can also evaluate stereotypic, emotional, and cognitive behaviors in humans, such as the motor rituals of patients with obsessive-compulsive disorder (OCD), executed and recorded at home, in natural conditions (Zor et al., 2009). The challenge of studying co-pathologies can also be appreciated when considering the strong association between Alzheimer’s disease and epilepsy (Alves et al., 2022, 2023a,2023b).

The landscape of the epilepsies and neuropsychiatric comorbidities is also shaped by a highly integrated construction of cellular and molecular elements, initially morphodynamically defined, followed by associations with increasingly complex features and their emergent functions as epileptogenic networks at the microscale (Szabo et al., 2015). Therefore, “while the recent shifts in focus to the microscale mechanisms enabled by high-resolution electrophysiological and optical imaging technologies have revealed unexpected levels of complexity, such new insights into microcircuit organization under healthy and pathophysiological conditions could also lead to a higher degree of specificity in targeting epilepsy, as well as to more individualized treatment” (Szabo et al., 2015). Mossy fiber sprouting has long been recognized as a critical factor in hippocampal hyperexcitability and dentate granule cell dysfunction associated with temporal lobe epilepsy (Sloviter, 1985; Sloviter et al., 2006). Tu et al. (2026) explored mossy fiber sprouting in resected hippocampi (*n* = 20) from patients with mesial temporal lobe epilepsy (MTLE) compared to *postmortem* controls (*n* = 20), using alpha-smooth muscle actin (αSMA) and reference markers for pathological cross-validation. The findings suggest the possibility of long-range mossy fiber sprouting associated with a regionally detected hypermetabolic state in the hippocampus of patients with drug-resistant temporal lobe epilepsy. Nevertheless, deep and superficial pyramidal cells form functionally distinct sublayers in the CA1 hippocampal field for theta oscillations (Mizuseki et al., 2011), and further steps must be taken to bridge the gap between the micro- and the macroscale, as follows: “ostensibly similar epilepsy expression at the macroscopic scale can originate from a variety of mechanisms at the microscopic scale. Thus, a detailed understanding of seizure mechanisms at the microscale, encompassing cellular signaling and communication, is necessary to explain epilepsy expression at the macroscale, including seizure behavior, EEG patterns, and neuroimaging findings” (Farrell et al., 2019).

Dendritic spine abnormalities are found in animal models of epilepsy and in patients, which may relate to dendritic pathology and hyperexcitable circuits and seizures—difficult to differentiate their effects on seizures versus other comorbidities—or seizures may cause damage to dendrites and spines, contributing to progressive epileptogenesis (Wong and Guo, 2013). Besides altered dendritic morphology and spine loss in hippocampal neurons, long-lasting epilepsy and seizure recurrence *per se* may not unavoidably produce dendritic pathology in human cerebral cortices derived from epilepsy surgery (Rossini et al., 2021). In the cases of dendritic remodeling, the study by Schunemann et al. (2025), from approximately 4,000 morphologically reconstructed human dendritic spines across different ages, gender, and tissue conditions, used a deep learning algorithm for three-dimensional spine reconstruction, useful for revealing connections between spine morphology, brain function, and the mechanisms of neurological and neuropsychiatric comorbidities (see also Guerra et al., 2023; Renner and Rasia-Filho, 2023).

Owing to technical advances, Chen R. et al. (2021) used channelrhodopsin ChRmine for transcranial photoactivation of defined neural midbrain and brainstem circuits, at unprecedented depths (up to 7 mm), with millisecond precision, achieving behavioral modulation without surgery and enabling implant-free deep brain optogenetics. These manipulations can be performed using closed-loop feedback control for seizure suppression, thereby avoiding the detrimental side effects of continuous stimulation (Berényi et al., 2012). In the intrahippocampal kainic acid model of temporal lobe epilepsy, Krook-Magnuson et al. (2013) optogenetically targeted hippocampal parvalbumin (PV)^+^ inhibitory (GABAergic) interneurons providing feedforward inhibition and cessation of focal seizures in wild-type animals. As demonstrated, “local and systemic injections can be used for effective viral delivery to the brain. With local injections, one could possibly ameliorate focal epilepsy, prefrontal cortex dysfunction or hippocampal memory disorders. By contrast, systemic introduction of virus could be used in contexts where global interventions are necessary, for instance, to correct generalized seizures, or for psychiatric and neurodegenerative disorders” (Vormstein-Schneider et al. (2020). Indeed, multi-scale investigation tools of seizure networks currently involve adeno-associated virus-mediated, optogenetic reductions in network firing rates of human hippocampal slices recorded on high-density microelectrode arrays under several hyperactivity-provoking conditions (Andrews et al., 2024).

Moreover, in the context of scaling from molecules, connectomics, complexity, and emergent functions, two recent studies are relevant for tools such as deep/machine learning and “virtual twins.” First, Lepeu et al. (2024) state that the predictability of critical transitions from normal to epileptic activity represents a major challenge, but theory predicts the appearance of subtle dynamical signatures near instability. These authors verified that predictions on bifurcations applied to the onset of hippocampal seizures and provided concordant results from *in silico* modeling, optogenetics experiments in male mice and intracranial EEG recordings in human patients with epilepsy. Second, through simulations of a conductance-based neuronal network model that reproduces spontaneous seizure-like events, Souza et al. (2026) identified that slow K^+^ channels play an important role in seizure generation. The key finding is the consistent presence of a prolonged period of neuronal silence in human electrophysiological data that precedes seizure onset, establishing it as a physiologically relevant biomarker for seizure prediction. A simulated targeted suppression strategy significantly shortened long seizure durations by up to 93%, confirming its enormous value for prediction and neuromodulation. Indeed, artificial intelligence and data-driven science, deep and machine learning techniques, computer vision, among others, now enable a whole new range of approaches for the analysis of large multimodal datasets, including neuroimaging, electrophysiology, genetics, and behavioral data (Cota and Garcia-Cairasco, 2026).

### 5.1 Approaching epilepsy as a complex system with functional emergent properties

Complex systems are involved in abnormal functioning and the refractoriness to pharmacological approaches of such neural circuits (Garcia-Cairasco, 2009a). There are current concerns for the definition of pharmacoresistant epilepsy with multilevel complexity, its pathophysiology, and its treatment when it is associated with comorbidities (Servilha-Menezes and Garcia-Cairasco, 2022). New integrative approaches applied to pharmacoresistant epilepsy are presented below.

Epilepsy is defined as pharmacoresistant when there is a lack of response to two or more tolerated and appropriately chosen antiseizure medications (ASM). In such cases, after application of the International League Against Epilepsy (ILAE) classification (Beniczky et al., 2025), neurosurgery may be recommended to remove the “*epileptogenic zone*” (Bancaud, 1980). It is worth noting that neurosurgical procedures based upon a variety of “localizationist-like” definitions of focus/epileptogenic zone are a subject of contemporary debate (Anderson, 2021). The continuous introduction of new therapeutic possibilities, including several powerful neurotechnologies such as vagus nerve stimulation, neuromodulation, closed loops, direct or transcranial magnetic stimulation, has helped to propose therapeutic paradigm shifts (Ryvlin et al., 2021). More advanced techniques use the combination of several tools to predict the specific regions or networks that are more sensitive to blockade or stimulation (e.g., by thermocoagulation or chemogenetics). Hopefully, they might also be targets of nanotechnology with controlled/safe, non-invasive optogenetics in the near future (Chen R. et al., 2021), in addition to optogenetics in human *in vitro* preparations/slices (Andrews et al., 2024). One example of these current approaches was provided by Vetkas et al. (2022) when pinpointing specific regions in the normative epilepsy deep brain stimulation (DBS) network: “Graph network analysis was used to describe the relationship between regions in the identified network. Furthermore, we examined the associations of the epilepsy deep brain stimulation network with disease pathophysiology, canonical resting state networks, and findings from a systematic review of resting state functional MRI studies in epilepsy deep brain stimulation patients.”

The concept of “epileptogenic zone” identified by stereo-electroencephalography (SEEG) has been notably useful for the more recent proposal by Bartolomei’s group (Balatskaya et al., 2020) regarding a network of hyperexcitable connected regions generating seizures and secondarily leading to ictal spreading in propagation networks, consequently giving rise to what was named the “Connectivity Epileptogenicity Index.” On the other hand, a crucial step forward will be the recognition of the complexity of the description/definition of the neural networks and their response to DBS. Currently, including complex systems and the characterization of emergent functions in epilepsy research and comorbidities is an obligatory step toward an integrative scenario.

In addition to the approaches mentioned above, other research avenues to the neuroglial morphology and pathophysiology of epilepsy involve studies with experimental animals, *ex vivo* experimental animal or human slices, cell cultures, organoids, and computational models. Each of these approaches provides a huge amount of information at all scales, but epilepsies and comorbidities together have a complexity that grows (for a comprehensive review of the concept of complexity in the epilepsies and comorbidities, particularly associated with pharmacorresistance, see Servilha-Menezes and Garcia-Cairasco, 2022). To further integrate approaches, both the structural and the functional aspects of the brain in epilepsies need to be taken as a “huge puzzle” (Garcia-Cairasco, 2009b). That is because, as proposed by Kuhn (1962), science advances with problem-puzzling strategies, which represent the way to remove dogmas in research and produce the so-called “scientific revolutions” by means of progressive, detailed, summatory exercises of creative work. For a comprehensive review and the proposal of a paradigm shift in epilepsy research and its associated comorbidities, putting critically together ancestral knowledge and contemporary neuroscientific and neurotechnological advances, see Garcia-Cairasco et al. (2021).

Let us, then, introduce relevant research data derived from the use of epilepsy animal models and how they have helped to unravel crucial points to disentangle and further explore the morphodynamics of epilepsy in a complex system. For example, quantitative neuroethological studies evidenced complex sequences of behaviors (dyadic interactions) in very stereotyped flowcharts, where regular (control behaviors) showed to be statistically different compared to those expressed by genetically-selected epileptic animals (Garcia-Cairasco et al., 1992, 1996). Such valuable model is the Wistar Audiogenic Rat (*WAR*) strain (Doretto et al., 2003; Garcia-Cairasco et al., 2017), available to the Brazilian scientific community as *WARspf* animals (produced at the Multidisciplinary Center for Biological Research in the Area of Laboratory Animal Science (CEMIB) at the Universidade Estadual de Campinas (UNICAMP) and to the international scientific community after a Material Transfer Agreement (MTA) signed between the University of São Paulo/Brazil and the “Rat, Resource and Research Center”/University of Missouri/United States^2^ (RRRC Mutant Rat Strain- W/LnneRrrc. RRRC#: 00697). The value of sharing data, knowledge, infrastructure, has been highlighted by Bower (1996) in his bright questioning “What will save Neuroscience”: “For our field to advance, we must move from a point of view in which each of us is working with our own personal, unquantified, and therefore largely untestable model of how “our” part of the brain works, to shared, quantifiable testable models. It is my view that realistic neuronal models are ideal for this purpose.”

As examples of the contrast between neuroplastic changes associated with different models of epilepsy, the pilocarpine-induced *Status Epilepticus* (SE) model shows known hallmarks such as neurodegeneration (Castro et al., 2011) and mossy fiber sprouting (Kandratavicius et al., 2014). Conversely, in kindled (repeated) audiogenic seizures in WARs, although behavioral (Garcia-Cairasco et al., 1996) and EEG (Dutra Moraes et al., 2000) evidence of forebrain recruitment, there is neither exuberant hippocampal cell loss (as seen in SE) nor mossy fiber sprouting (Galvis-Alonso et al., 2004) in a previously brainstem-dependent model of tonic-clonic seizures (Garcia-Cairasco et al., 1996). In a recent publication, in the WAR strain (Placido et al., 2026), a genetically developed model, highly characterized in terms of complex epilepsy and comorbidities phenotypes, altered expression of P-glycoprotein was detected, strongly suggesting the WARs as a suitable model to study epilepsy pharmacoresistance (Placido et al., 2026).

Interestingly, human behavioral seizures (similar to those in animals) also follow statistical and probabilistic rules, as seen in cases of temporal or frontal lobe seizures in epileptic patients (Dal-Cól et al., 2006; Bertti et al., 2010). Patients were tested against a hypothesis previously proven using the WAR strain, involving both acute and chronic seizures (Garcia-Cairasco et al., 2017; Lazarini-Lopes et al., 2023) as well as SE induced by systemic and intra-hippocampal pilocarpine (Furtado et al., 2002; Castro et al., 2011). Data analyses evolved from the use of quantitative neuroethological studies (flowcharts and probabilistic χ^2^ dyadic behavioral interactions) in animal and clinical models (Dal-Cól et al., 2006; Garcia-Cairasco et al., 2009; Bertti et al., 2010; Garcia-Cairasco et al., 2017) to the use of complex systems measurements. Complex systems measurements, such as those in graphs, use visual/statistical tools, including betweenness centrality, clusterization indexes, and local and regional entropy, among others. Results showed that, when behavioral sequences of temporal and frontal lobe seizures are compared, they are significantly different; for example, when contrasting automatisms (temporal lobe) with motor tonic-clonic seizures (frontal lobe), it is evident that the underlying substrates are quite different (Tejada et al., 2013).

Furthermore, the relevance of quantitative behavioral studies in epilepsy, as highlighted by Krakauer et al. (2017) in *Neuroscience Needs Behavior*, is reinforced by the concept of computational neuroethology (Datta et al., 2019). This approach aims to characterize behavioral sequences of multiple simultaneous animals recorded via video, utilizing DeepLabcut (Mathis et al., 2018) and MoSeq (Wiltschko et al., 2015) software in experimental models of epilepsy, where behaviors are revealed through syllables, or grammar in microseconds.

In the WAR strain model mentioned above, flowcharts and graphs analysis could be compared for the hypergrooming behavior induced by microinjection of oxytocin into the amygdaloid complex, a model of stereotyped compulsion (Marroni et al., 2007; Marroni et al., 2025). Together, these data indicate that quantitative results reveal aspects of epilepsies not previously reported. Current quantitative tools offer high resolution, allowing, for example, the recognition of an “epileptic phenotype” solely through behavioral analysis without EEG recording or, as stated by Gschwind and Soltesz (2024) and Gschwind et al. (2023), the unbiased detection of hidden inter-ictal and ictal behavioral phenotypes. The concept of “*hidden behavioral fingerprints in epilepsy*” begins to overcome this bottleneck, advancing toward unbiased assessment approaches for the epilepsies and future integrated, theoretical, and experimental approaches. Concurrently, the application of mathematical procedures enables the linkage of complex systems neuroscience with experimental data, providing multi-scale results on models organized around structure, function, and behavioral display in both normal and pathological conditions.

As a natural consequence of advances in basic physiological concepts and their human neurological substrates—including Penfield’s homunculus, Scoville’s hippocampal removal, and Brenda Milner’s description of consequent declarative memory loss and preserved procedural memory in epilepsy patients—epilepsy patients can now be studied and treated using new integrative approaches that consider complex systems organization (Garcia-Cairasco, 2009a). Although achieving high-level morphological detail for all neurons is difficult, we propose that the morphodynamics of neurons and glia in neural circuits can be studied as an initial step toward understanding proper wiring and stable properties—including neurotransmitters, neuromodulators, receptors, and intracellular signal transduction components—as well as plastic features, environmental stimuli, attention, and learning. Using various current approaches, such data can be assembled ranging from developing and adult human neurons (Petanjek et al., 2023; Kostović, 2024), human organoids and assembloid models (Antunes et al., 2025; Li and Knoblich, 2025; Perets et al., 2026; Tenreiro and Muotri, 2026), and experimental animal models in normal or pathological conditions (Tejada et al., 2013; Martin et al., 2022), to network-level features, computational simulations, neuroengineering, and artificial intelligence (Oruro and Pardo, 2025; Cota and Garcia-Cairasco, 2026) for further predictions and research hypotheses.

## 6 Final remarks and perspectives

We present morphodynamics as a unifying framework to integrate neuronal form, function, and behavior across multiple levels of organization, from cellular processes to complex behavioral and pathological phenomena. Morphology remains a fundamental step toward understanding the functions of neural cells and how network architecture shapes them (Cajal, 1909–1911; DeFelipe, 2011; Renner and Rasia-Filho, 2023; Shapson-Coe et al., 2024; The MICrONS Consortium, 2025; Renner et al., 2026a,b). We propose that neuronal morphology, understood as an emergent and continuously evolving level of organization, is not merely a substrate of function but an active component in the generation of emergent properties in complex systems. Current neuroscience approaches can benefit from further integrating morphodynamic perspectives, neural network emergent functions, and other complementary complex systems perspectives to understand the broad organization of social behaviors in animal models and in humans. For example, different hippocampal dendritic arbor intrinsic properties and integrative compartments, single-dendritic plateau potentials, synaptic weight modulation and plasticity, neuronal microcircuit function, and population activity over behavioral timescales collectively contribute to Behavioral Timescale Synaptic Plasticity (BTSP), a neuronal substrate that has been implicated in adaptive, experience-based learning and memory that can improve behavior (Magee, 2026). Moreover, BTSP computational models may help integrate this biological mechanism into large-scale neural simulation and artificial intelligence frameworks (Madar et al., 2025).

There might be evolved similarities as well as species-specific characteristics in morphodynamics. For some deep areas of the human brain, 3D reconstructions of neurons or immunohistochemical studies are the only available baseline morphological data from *postmortem* brain samples thus far (e.g., for the deep amygdaloid nuclei, Sorvari et al., 1996; Guerra et al., 2023; Vásquez et al., 2024). For the human cerebral cortex, morphological, electrophysiological, and/or transcriptomic findings are available from tissue removed during neurosurgeries (e.g., Allen Brain Atlas data portal at https://celltypes.brain-map.org/; Chartrand et al., 2023; Shapson-Coe et al., 2024; Planert et al., 2025). All these data are crucial to understanding the human brain cellular evolution and connectome and, consequently, how the temporospatial integration of information processing can generate emergent properties in our complex nervous system and the organized functions it supports.

Hence, morphodynamics can be linked to (1) transcriptomic and functional characterizations of specific neuronal subpopulations (Hodge et al., 2019; Kalmbach et al., 2021) –although gene expression profiles can vary “in individual cells of a type, without losing their cell type identity” (Xu et al., 2018) or after synaptic plasticity and changes in social environment (Milewski et al., 2025); (2) the identification of the molecular composition of the neural cells and their diversity, based on multiple neurochemical markers (McDonald, 2023); and (3) electrophysiological recordings (Planert et al., 2025). The structure, fine-scale synaptic connections, and functional characterization of coexisting neuron subpopulations (Ding et al., 2025) may compose a brain-wide map of neural activity during complex behavior display (International Brain Laboratory et al., 2025). This integration will serve to address emergent properties associated with multi-modal magnetic resonance images of complex, parceled areas, neural networks, and their functional implications (Glasser et al., 2016; Kolb and Whishaw, 2021; Schimmelpfennig et al., 2023).

Our data on early attachment and mother-infant interactions reinforce the need to move toward an integrative complex systems perspective. While many experimental approaches necessarily focus on one variable at a time, computational models provide a means to integrate data across multiple levels of organization, allowing the exploration of how molecular and cellular changes shape neural circuit dynamics and, in turn, behavioral processes. Importantly, such models also make it possible to examine reciprocal influences across levels, in which higher-level interactions can modulate lower-level processes, giving rise to emergent properties at each level of organization. This integrative approach reveals dynamic relationships that may not be accessible through isolated experimental manipulations and offers a practical framework for linking cellular mechanisms, circuit function, and behavior. By combining computational modeling with empirical data, it may be possible to identify general developmental principles, generate testable hypotheses, and investigate mechanisms operating across multiple temporal and spatial scales. From this perspective, approaches inspired by morphodynamics provide a promising avenue to examine how form, function, and experience interact over development, deepening our understanding of how early mother-infant relationships shape developmental trajectories and exert lasting influences across the lifespan.

In epilepsy research, the next logical step is to use computer vision and deep learning to characterize seizures from videos in clinical settings, allowing for quantitatively and automated evaluation of the semiology of the seizures (Ahmedt-Aristizabal et al., 2024). SEEG data from drug-resistant focal epilepsy patients (prefrontal cortex at seizure onset) can then be associated with scored semiological features to link clinical manifestations with network analysis of ictal brain activities (Gauld et al., 2025). This is a refinement of previous work utilizing neuroethological tools—including flowcharts, graph analysis and Markov’s chains evaluations—in both experimental epilepsy models (Garcia-Cairasco et al., 1996; Lazarini-Lopes et al., 2023, Marroni et al., 2025) and in patients with epilepsy presenting temporal and frontal lobe seizures (Dal-Cól et al., 2006; Bertti et al., 2010; Bertti et al., 2014).

Furthermore, in pharmacoresistant epilepsy patients, the concept of virtual twins (Wang et al., 2025; Hashemi et al., 2025; Ziaeemehr et al., 2025) provides a novel, comprehensive platform for *in silico* simulation of the most efficient neurosurgical approach and strategy for individual patients before surgery is performed. The concept of virtual twins has also been applied recently to the study of brain tumors (Meghdadi et al., 2026), a well-known cause of epilepsy (Aronica et al., 2023). Additionally, The Virtual Brain has been coupled to the Networks using Python and NEURON (NetPyNE) tool as a way to interface biophysical circuits with whole-brain networks (Romaro et al., 2021; Bragin et al., 2024) and complex system functioning. Indeed, comparing experimental and clinical settings, the behavioral expression of epileptic seizures obeys complex system rules (Garcia-Cairasco, 2009a,b; Tejada et al., 2013). Following the progressive expression of seizures, plastic neuroanatomical, neurochemical, and electrophysiological changes occur, which are the substrate for additional behavioral changes and the emergent functions of the epileptogenic network. Pharmacoresistance can be a consequence of these events, largely because of hidden or newly developed comorbidities anchored in hypercomplex networks, rather than appearing as isolated events (Servilha-Menezes and Garcia-Cairasco, 2022). From morphodynamics to functional complexity, we are merging parts to form a continuum within a larger entity with emergent properties (artistically illustrated in Figure 1B). The integration of all levels of experimental and theoretical approaches with current neurotechniques, particularly those associated with computational modeling, deep and machine learning, emphasizes the complexity of the current challenges and, thus, the complexity of the solutions needed (Tejada et al., 2013; Garcia-Cairasco et al., 2021; Murphy et al., 2022). Previous frameworks have explored emergence at the level of large-scale neural networks and conscious processing, emphasizing distributed activity and neuromodulatory regulation (e.g., Changeux and Lou, 2011). In this context, artificial intelligence and artificial neural networks may offer useful complementary tools to investigate how morphological heterogeneity influences information processing across cortical hierarchies, helping to link neuronal morphology, connectivity, and emergent functions in the complex nervous system.

To conclude, aiming for potential translational implications for clinical and therapeutic approaches, we encourage collaborative work through multi-institutional e-teams in the framework of the Complex System Digital Campus, a UNESCO UniTwin program to collaboratively address these current open issues in neuroscience and complex systems. Moving forward with morphodynamics, we can develop shared tools for analyzing multi-source datasets ranging from the unit to the systemic level, covering both normal and pathological conditions. Advancing a morphodynamic perspective in neuroscience may contribute to bridging current gaps between structure, function, and behavior, providing a coherent framework to investigate how complex neural systems develop, adapt, and give rise to emergent properties. This framework opens new avenues for understanding both normal brain function and its alterations in disease, integrating empirical data with computational and theoretical approaches.

## Footnotes

1
https://www.cs-dc.org/

2
https://www.rrrc.us/
